# Sensory Detection by the Vomeronasal Organ Modulates Experience-Dependent Social Behaviors in Female Mice

**DOI:** 10.3389/fncel.2021.638800

**Published:** 2021-02-17

**Authors:** Anne-Charlotte Trouillet, Chantal Moussu, Kevin Poissenot, Matthieu Keller, Lutz Birnbaumer, Trese Leinders-Zufall, Frank Zufall, Pablo Chamero

**Affiliations:** ^1^Laboratoire de Physiologie de la Reproduction et des Comportements, UMR 0085 INRAE-CNRS-IFCE-University of Tours, Nouzilly, France; ^2^Neurobiology Laboratory, National Institute of Environmental Health Sciences, National Institutes of Health, Durham, NC, United States; ^3^School of Medical Sciences, Institute of Biomedical Research (BIOMED), Catholic University of Argentina, Buenos Aires, Argentina; ^4^Center for Integrative Physiology and Molecular Medicine, Saarland University, Homburg, Germany

**Keywords:** olfactory, Gαi2, maternal, lordosis, sex preference, kisspeptin

## Abstract

In mice, social behaviors are largely controlled by the olfactory system. Pheromone detection induces naïve virgin females to retrieve isolated pups to the nest and to be sexually receptive to males, but social experience increases the performance of both types of innate behaviors. Whether animals are intrinsically sensitive to the smell of conspecifics, or the detection of olfactory cues modulates experience for the display of social responses is currently unclear. Here, we employed mice with an olfactory-specific deletion of the G protein Gαi2, which partially eliminates sensory function in the vomeronasal organ (VNO), to show that social behavior in female mice results from interactions between intrinsic mechanisms in the vomeronasal system and experience-dependent plasticity. In pup- and sexually-naïve females, Gαi2 deletion elicited a reduction in pup retrieval behavior, but not in sexual receptivity. By contrast, experienced animals showed normal maternal behavior, but the experience-dependent increase in sexual receptivity was incomplete. Further, lower receptivity was accompanied by reduced neuronal activity in the anterior accessory olfactory bulb and the rostral periventricular area of the third ventricle. Therefore, neural mechanisms utilize intrinsic sensitivity in the mouse vomeronasal system and enable plasticity to display consistent social behavior.

## Introduction

The onset of female-specific behaviors in mice results from interactions between sensory detection mechanisms and plastic neuronal pathways. Rapid responses in females’ behaviour—such as pup care (Lévy et al., 2004), or sexual receptivity to males (Keller et al., 2009)—start with the detection of chemical signals by the olfactory system. Although these olfactory-driven responses are typically hard-wired, aspects of female behavior may evolve after social experience. For example, virgin females recognize pup odors and retrieve isolated pups to the nest to some extent, but maternity greatly increases this type of parental behavior (Stolzenberg and Rissman, 2011; Kohl et al., 2017). Similarly, the display of sexual receptivity in females, indexed by the incidence of lordosis stance, largely depends on the detection of olfactory signals by the vomeronasal organ (VNO) (Keller et al., 2006; Oboti et al., 2014; Hellier et al., 2018) and is considerably enhanced by experience (Thompson and Edwards, 1971; Bonthuis et al., 2011). Plastic changes in central brain areas have been shown to modulate instinctive olfactory-mediated female behaviors (Stowers and Liberles, 2016), but whether sensory detection by the olfactory system also influences social experience remains unexplored.

Pheromones play a key role in the regulation of innate reproductive responses in female mice and are largely detected by sensory neurons in the VNO (Chamero et al., 2012; Stowers and Kuo, 2015; Ishii and Touhara, 2019). Genetic ablation of Trpc2, the primary signal transduction pathway of VNO neurons, results in several behavioral deficits in mice, such as display of sexual behavior toward both males and females indiscriminately, lack of maternal aggression in females as well as enhanced pup care in males (Leypold et al., 2002; Stowers et al., 2002; Kimchi et al., 2007; Hasen and Gammie, 2009; Wu et al., 2014). We have previously shown that the G protein Gαo is necessary for sensory function of basal vomeronasal sensory neurons (VSNs), maternal aggression, and lordosis behavior in female mice (Chamero et al., 2011; Oboti et al., 2014). By contrast, apical VSNs, which express Gαi2 and V1R receptors, are critical for pup-directed aggression and parental care in males (Trouillet et al., 2019) and have also been associated with maternal aggression (Del Punta et al., 2002; Norlin et al., 2003). This suggests that both apical and basal vomeronasal pathways control female-typical behaviors activating certain behaviors and repressing others. However, the neural VNO mechanisms involved and the role of social experience in controlling the display of female-typical behaviors remain largely unknown.

To uncover the neuronal VNO substrate leading to specific female behaviors, we used female mice with a conditional knockout of Gαi2, in which the apical half of the VNO lacks sensory function (Trouillet et al., 2019). We used two different behavioral paradigms, pup retrieval, and sexual receptivity, in which female mice were naïve to the stimuli, either a mouse infant or an adult male. Mutant females showed reduced pup retrieval behavior, whereas sexual receptivity was not different from control mice in the initial tests. Then, we evaluated the effect of social experience in the display of these behaviors. Remarkably, Gαi2 mutant females displayed normal pup retrieval behavior and maternal aggression after parturition, while sexual experience was not sufficient for Gαi2 mutants to reach control levels of sexual receptivity. These results demonstrate the role of V1R and Gαi2+ neurons in the display of lordosis and detection of pup cues. Unexpectedly, social experience in Gαi2 deficient mice leads to divergent effects on behavior: improvement of pup retrieval without any beneficial effects on the acquisition of high lordosis performance. Altogether, our data suggest that the detection of pheromones by the VNO influences olfactory-mediated behavior in females after social experience, although with distinctive traits for different behaviors.

## Materials and Methods

### Mice

Animal care and experimental procedures were performed following French and European guidelines on the protection of animals used for scientific purposes and approved by an ethical committee for animal experimentation (CEEA Val de Loire project 12785). Mice were kept under standard 12 h light/dark cycles with food and water *ad libitum*. cGαi2^–/–^ and cGαi2^+/–^ mice were generated as described (Trouillet et al., 2019). Briefly, floxed *Gnai2* (*Gnai2*^fx/fx^) mice with mixed 129sv × C57BL/6 background were crossed with mice carrying a transgene directing the expression of Cre recombinase under the control of the OMP promoter (*Omp*-Cre mice; B6; 129P2-*Omp*^tm4(cre)Mom^/ MomJ; The Jackson Laboratory, JR# 006668; backcrossed into C57BL6/J for 8 generations; neomycin cassette is absent). *Gnai2*^fx/fx^ mice carry loxP sites inserted into the introns that flank exons 2 and 4. Breeding established offspring that were homozygous for the floxed *Gnai2* alleles and heterozygous for Cre and *Omp* (*Gnai2*^fx/fx^ *Omp*^cre/+^ or cGαi2^–/–^). In these mice, Cre-mediated *Gnai2* deletion was restricted to *Omp*-positive cells. Animals heterozygous for both alleles (*Gnai2*^fx/+^ *Omp*^cre/+^ or cGαi2^+/–^) served as controls. Adult females (more than 8 weeks) were used except specified otherwise. Adult (8 weeks or older) C57BL/6 males and females (Janvier Labs) and Balb/c males (Janvier Labs) were used as stimulus animals.

### Surgery

Stimulus females used for the mounting behavior assay and experimental females used in lordosis, habituation-dishabituation, and olfactory preference tests were ovariectomized under general anesthesia (xylazine 10 mg/kg, ketamine 100 mg/kg), implanted with a SILASTIC capsule filled with estradiol-benzoate (1:1 mix with cholesterol; Sigma-Aldrich). Females were allowed to recover from surgery at least 2 weeks before testing. Females were subcutaneously treated with progesterone (1 mg/100 μl in sesame oil, Sigma-Aldrich) to induce a pharmacological estrus state 4 h before each test. Juvenile (P18-22) C57BL/6 stimulus males were castrated under isoflurane general anesthesia and used in adulthood.

### Behavior

Mice were moved to the experimental room 2 h before testing. Assays were conducted 2 h before the start of the dark period. Experiments were videotaped and subsequently analyzed by a blind experimenter.

#### Parental Behaviors in Virgin Females

Sexually naïve females (*N* = 12 of each genotype) were tested for behavior in the presence of an alien 1–2 day old C57BL/6 (wild type) neonate introduced into the home-cage of the test mouse for 10 min. Females were individually housed for 1 week before testing. To minimize handling and stress to the pups, one single pup was taken from the nest immediately prior to the test avoiding prolonged exposure to cold. Mice were categorized as parental if they retrieved a pup into their nest, and otherwise as neutral. We did not observe aggressive behavior. Latency to retrieve a pup, pup grooming, and nesting times were scored.

#### Maternal Aggression

Sexually naïve females (20 cGαi2^+/–^ and 16 cGαi2^–/–^) were paired with a C57BL/6 male for 1 week. Bedding and nesting materials were changed before parturition and left until the end of the experiments. On postnatal days 2–4, females were tested daily for maternal aggression using the resident—intruder paradigm (Chamero et al., 2011). Before testing, pups were placed in a box next to their mother’s cage to avoid potential injuries by the intruder. Testing lasted 10 min and began when a sexually inexperienced intruder (Balb/c adult male, group-housed) was placed in the home cage of the test female mouse (resident). Residents were not exposed twice to the same intruder. Aggressive behavior was defined as lunging, biting, chasing, tail rattling, wrestling, and kicking. Attack bouts were defined as a succession of aggressive events separated by <3 s. Proportion of females attacking the intruder, latency to attack, and cumulative attack duration and episodes of 3 consecutive tests were scored.

#### Pup Retrieval

After maternal aggression, the same females were tested for retrieval of their own pups. On postnatal day 5, five pups were randomly dispersed at the opposite side of the nest. The latencies to retrieve each pup to the nest were scored.

#### Olfactory Habituation Dishabituation Assay

Sexually naïve females (10 cGαi2^+/–^ and 9 cGαi2^–/–^) were evaluated for their ability to distinguish urine sources. Females were ovariectomized, supplemented in estradiol, and subcutaneously treated with progesterone 4 h before the experiment. The test was conducted in the female’s home-cage and an odor stimulus was placed on a cotton-tipped applicator through the hole of the cage lid. Direct contact with the applicator was allowed. Food and water were removed from the grid and mice were allowed to familiarize themselves with a clean applicator for 30 min before the test. The test started with a first exposition for 1 min to distilled water (10 μl). This procedure was repeated three times with 1 min intervals, followed by a single presentation of intact male urine (10 μl, collected and pooled from 4 C57BL/6 group-housed adult mice). The time spent sniffing in close contact with the applicator was measured for each presentation.

#### Olfactory Preference Test

Sexually naïve females (10 cGαi2^+/–^ and 9 cGαi2^–/–^) were tested for their male-directed odor preference in a Y-maze apparatus as described previously (Jouhanneau et al., 2014). Females were ovariectomized, supplemented in estradiol, and subcutaneously treated with progesterone 4 h before the experiment. Two days before the test assay, mice were accustomed to the maze for 9 min in absence of an odor stimulus. On test day, urine from two distinct sources were placed in the arms’ ends of the Y-maze on a filter paper in a plastic weigh boat behind a perforated wall. The apparatus was cleaned with 20% ethanol between subjects. Each animal was tested twice: first, with either intact male or estrous female urine; and second, with either intact or castrated male urine. Equal urine volumes from 4 C57BL/6 animals per condition were pooled and the estrus status of female donors was assessed by vaginal cytology. The time of chemosensory investigation was recorded. Preference scores were calculated as the difference between times spent on each urine source over total time of chemosensory investigation.

#### Mounting Behavior

Sexually naïve females (20 cGαi2^+/–^ and 17 cGαi2^–/–^) were individually housed for 1 week before testing. Testing lasted 10 min and began when a sexually inexperienced intruder (either male, female in pharmacological estrus, or castrated male, group-housed) was placed in the home cage of the test mouse (female resident), whose bedding had not been changed for at least 4 days. Each female was tested with every intruder in a randomized order. The proportion of females mounting the intruder was recorded.

#### Female Sexual Receptivity (Lordosis)

Sexually naïve females (10 cGαi2^+/–^ and 9 cGαi2^–/–^) were tested for their sexual receptivity toward male mounting. Females were ovariectomized, supplemented in estradiol, and subcutaneously treated with progesterone 4 h before the experiment. The test began when the female was introduced in the home cage of a sexually experienced C57BL/6 stud male and lasted for 20 min or until the female received 20 mounts. Stud males were previously trained, and only males showing a mounting latency of less than 5 min were used. Females were not exposed twice to the same stud male. Females were tested three times with a 4 days interval between tests. Lordosis was scored when the female arched her back, lift her tail and adopted a rigid posture standing on all four paws, independently of whether the male was able to achieve intromission. The lordosis quotient (number of lordosis responses/number of mounts) was scored.

### Immunostaining

#### Tissue Preparation

90 min after sexual behavior or interaction with a neonate, mice were anesthetized by an overdose of pentobarbital (Ceva) and perfused transcardially with 0.9% saline solution followed by 0.1 M phosphate buffer (PB) containing 4% paraformaldehyde (PFA). Brains were removed, postfixed overnight in 4% PFA, and cryoprotected in 0.1 M PB containing 30% sucrose. Brain and olfactory bulbs (OB) were embedded separately in Tissue-Tek < *cps*:*sup* > ® O.C.T^TM^ compound, snap-frozen in cold isopentane, and processed on a Leica CM 3050S cryostat. Samples were cut in 30 μm serial free-floating sections (coronal for brains, sagittal for OB) using tris-buffered saline solution (TBS) containing 0.1% sodium azide.

#### c-Fos Immunolabeling

Sections were washed (3 × 5 min) in TBS, endogenous peroxidases were blocked for 30 min in TBS containing 3% H_2_O_2_. Sections were incubated in blocking solution (TBS containing 0.1% Triton X-100, TBS-T, and 5% donkey serum) 2 h at room temperature (RT), and overnight at 4∘C in blocking solution supplemented with the c-Fos primary antibody (1:500; rabbit polyclonal #sc-52, Santa Cruz Biotechnology). Sections were then washed in TBS and incubated in TBS-T supplemented with secondary antibody (1:1,000; biotinylated donkey anti-rabbit IgG, Jackson ImmunoResearch) for 2 h at RT. Signals were amplified with VECTASTAIN ABC kit (Vector) and visualized with diaminobenzidine (DAB 0,02%, 0,01% H_2_O_2_ in 0,05 M Tris, pH 7,4). Slides were mounted with DPX (Sigma-Aldrich).

#### Kisspeptin/c-Fos Immunolabeling

Sections were washed in TBS, incubated in blocking solution (TBS containing 0.1% Triton X-100, TBS-T, and 5% donkey serum) 2 h at RT, incubated 72 h at 4°C in blocking solution supplemented with c-Fos (1:500; rabbit polyclonal #sc-52, Santa Cruz Biotechnology) and kisspeptin [1:10,000; sheep polyclonal #AC053, generous gift of I. Franceschini (Franceschini et al., 2013)] primary antibodies. Sections were then washed in TBS and incubated in TBS-T supplemented with secondary antibodies (1:500; Cy3 donkey anti-rabbit-IgG and Alexa-488 donkey anti-sheep-IgG, Jackson ImmunoResearch) for 2 h at RT. Nuclei were counterstained with 4′,6-diamidino-2-phenylindole (DAPI; 0.5 μg/ml, Sigma-Aldrich) for 2 min. Slides were mounted with Fluoromount^TM^ (Sigma-Aldrich).

#### Analysis

For c-Fos experiments, slides were scanned using an automatic slide scanner (Axio Scan.Z1, Zeiss). Regions of interest were drawn based on the Paxinos mouse brain atlas using Zen (blue edition 3.0, Zeiss). The number of c-Fos+ nuclei was automatically counted bilaterally in these regions using the particle analyzer plug-in of Fiji. 2–3 images of the accessory olfactory bulb (AOB), 3 images of the rostral periventricular area of the third ventricle (RP3V), 2 images of the ventrolateral part of the ventromedial hypothalamic nucleus (VMHvl) and 4 images of the medial preoptic area (MPA) per animal were analyzed. Kisspeptin/c-Fos co-expression was quantified in 3–5 RP3V images/animal acquired on a Zeiss LSM-700 confocal laser-scanning microscope at 10× magnification. Images were manually analyzed in the entire *z*-axis with 3 μm step intervals.

### Reproductive Physiology

Young prepubertal females (17 cGαi2^+/–^ and 15 cGαi2^–/–^) were monitored daily for evidence of vaginal opening from postnatal day 15, and then for their first estrus by vaginal smears sampling. Briefly, vaginal smears were flushed with 15 μl of NaCl 0.9% solution and the estrus phase was identified by light microscopy after methylene-blue coloration of the smears. Adult females (47 cGαi2^+/–^ and 45 cGαi2^–/–^) were also examined daily for estrus status for 2 weeks using the same protocol. Adult females (61 cGαi2^+/–^ and 49 cGαi2^–/–^) were sacrificed during their diestrus or estrus phase to collect trunk blood and measure the weight of ovaries and uteri. Blood sera were obtained by centrifugation (2,500 × *g* for 25 min) and levels of progesterone were measured by immunoenzymatic assay. Assay sensitivity is 0.25 ng/ml. Sexually naïve females (76 cGαi2^+/–^ and 67 cGαi2^–/–^) were paired for 7 days with a C57BL/6 male. The proportion of pregnant females, the latency to deliver, and the number of pups born were collected.

### Statistics

Statistical analyses were performed using GraphPad Prism 9.0 (GraphPad Software, Inc.), and OriginPro 2016G (OriginLab Corporation). Assumptions of normality and homogeneity of variance were tested before conducting the following statistical approaches. Student’s *t*-test was used to measure the significance of the differences between two distributions. In case the results failed the test of normality, Mann-Whitney or Kolmogorov-Smirnov test was performed. Multiple groups were compared using a two- or three-way repeated-measures analysis of variance (ANOVA) with Bonferonni’s tests as *post hoc* comparison. Kruskal-Wallis test with Dunn’s multiple comparisons, or Friedman multiple comparisons in case of paired values, were used for non-normal distributions. Categorical data were analyzed with Fisher’s exact test, and correlations were assessed by the Pearson coefficient. The probability of error level (alpha) was chosen to be 0.05. Unless otherwise stated, results are presented as means ± SEM and documentation of individual data points.

## Results

### Inactivation of the Apical Vomeronasal Cell Layer Reduces Pup Retrieval in Virgin Females

VNO activity is necessary for the display of important pup-directed behaviors in male mice, such as pup-directed aggression (Tachikawa et al., 2013; Wu et al., 2014; Isogai et al., 2018; Trouillet et al., 2019), inhibition of sexual behaviors toward pups (Ferrero et al., 2013), and increased pup-retrieval and parenting behaviors (Wu et al., 2014; Trouillet et al., 2019). In virgin females, the role of the VNO in pup-directed behaviors is less clear. While some studies suggest that genetic ablation of VNO function in Trpc2^–/–^ females increase parenting behavior toward alien juveniles (Nakahara et al., 2016), this view is not supported by others (Wu et al., 2014).

**FIGURE 1:**
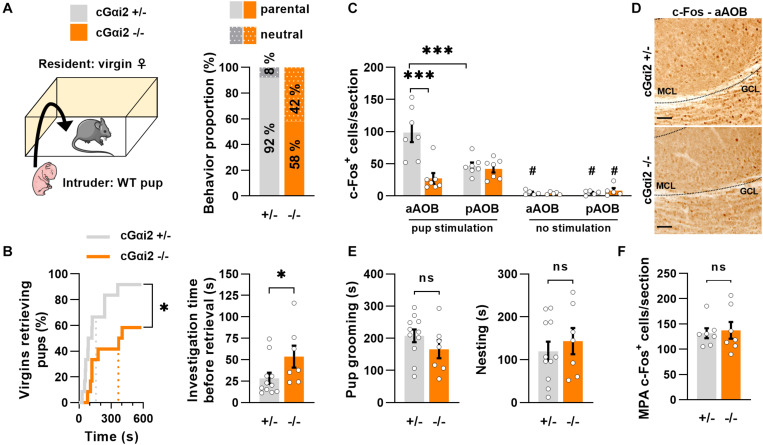
Pup retrieval is altered in virgin cGαi2^–/–^ females. **(A)** Comparison of pup-directed behavior of cGαi2^+/–^ vs. cGαi2^–/–^ virgin female residents (*N* = 12 per genotype). Wild type 1–2 day-old pups served as intruders. Females ignore (neutral) or retrieve neonates to their nest (parental). Females of both genotypes are predominantly parental (11 out of 12; 92% for cGαi2^+/–^ and 7 out of 12; 58% for cGαi2^–/–^). **(B)** *Left*, proportion of females retrieving pups over time (Kolmogorov-Smirnov test: *P* < 0.05). Dotted lines represent the mean latency. *Right*, time spent sniffing the pup before retrieval in cGαi2^–/–^ (*N* = 7) and cGαi2^+/–^ (*N* = 11) parental females (Mann-Whitney: *P* < 0.05). **(C)** Quantification of c-Fos+ cells per section of cGαi2^+/–^ and cGαi2^–/–^ virgin females (*N* = 7 per group) in control condition (no stimulation) and after exposure to an alien pup (pup stimulation) reveals a decreased c-Fos activation in the aAOB of cGαi2^–/–^ compared to cGαi2^+/–^ females, and a higher number of c-Fos+ cells in the anterior compared to the posterior part of the AOB of cGαi2^+/–^ females only [Three-way repeated measures ANOVA: (genotype) *F*_(__1, 12)_ = 6.719, *P* < 0.01; (region) *F*_(__1, 12)_ = 4.024, *P* = 0.068; (stimulation) *F*_(__1, 12)_ = 63.21, *P* < 0.001; (genotype × region × stimulation) *F*_(__1, 4)_ = 14.67, *P* < 0.05; Bonferroni *post hoc* tests: cGαi2^+/–^ aAOB vs. cGαi2^+/–^ pAOB and cGαi2^+/–^ aAOB vs. cGαi2^–/–^ aAOB *P* < 0.001; ^#^*P* < 0.05 vs. the stimulated equivalent condition; other comparison *P* = 0.3–0.9]. All three AOB layers were quantified. **(D)** Examples of c-Fos immunodetection in the aAOB of cGαi2^+/–^ and cGαi2^–/–^ virgin females exposed to an alien pup for 10 min. Scale bars, 25 μm. **(E)** The time spent grooming (*left*) and nesting (*right*) the neonates (*t*-test: *P* = 0.2–0.5). **(F)** Quantification of c-Fos^+^ cells per section in the MPA of cGαi2^+/–^ and cGαi2^–/–^ virgin females after exposure to an alien pup is not different (*t*-test: *P* = 0.8). AOB, accessory olfactory bulb (aAOB, anterior AOB; pAOB, posterior AOB); MCL, mitral cell layer; GCL, granular cell layer; MPA, medial preoptic area. *P < 0.05; ***P < 0.001.

To determine whether the vomeronasal pathway may partially regulate pup parenting in virgin females, we used mice carrying Olfactory Marker Protein (OMP)-dependent deletion of Gαi2 (cGαi2^–/–^), which impairs sensory signaling in apical VNO neurons (Trouillet et al., 2019). We exposed pup-naïve virgin females to 1–2 days old pups (Figure 1A) and the majority of females retrieved the pups to the nest, although the proportion of retrieving females was lower in cGαi2^–/–^ when compared to heterozygous controls (58 vs. 92%; Figure 1A). Measurement of the latency to retrieve pups showed that cGαi2^–/–^ virgin females retrieved pups with longer latency (*P* < 0.05; Figure 1B), even if they displayed more time sniffing the pups before retrieving (*P* < 0.05; Figure 1B). Next, we used c-Fos immunolabeling to confirm the role of vomeronasal signaling in pup detection by virgin females and examine whether pup odors were preferentially detected by either the apical or basal vomeronasal subsystems. We compared the density of c-Fos+ nuclei in all three layers of the accessory olfactory bulb (AOB), the first relay station of the pheromonal information transfer in the brain, after pup exposure in cGαi2^–/–^ and cGαi2^+/–^ virgin females (Figures 1C,D). After pup exposure, the density of c-Fos+ nuclei was nearly fourfold lower in the anterior part of the AOB of cGαi2^–/–^females (*P* < 0.001; Figure 1C), consistent with reduced sensory input from the apical VNO. Furthermore, we observed twofold more c-Fos+ nuclei in the anterior vs. posterior part of the AOB in control females (*P* < 0.001; Figure 1C), suggesting a preferential activation of apical Gαi2-expressing VSNs by pup odors. Once pups were retrieved to the nest, measures of parental care—pup grooming and nesting time—were not significantly different in cGαi2^–/–^ females (Figure 1E; *P* = 0.2–0.5). Consistent with this, the number of c-Fos+ cells in the medial preoptic area (MPA), a region involved in parenting (Numan, 1974), was also not significantly different in cGαi2^–/–^ females (Figure 1F). Together, these results indicate that Gαi2-dependent VNO inputs participate in pup odor detection in virgin females and that deletion of Gαi2 reduces pup retrieval, although pup-parenting behaviors are still displayed after retrieval.

### Deletion of Gαi2 Does Not Affect Maternal Behavior in Mothers

Both the main olfactory epithelium and VNO seem to be implicated in maternal behavior. Previous experiments using genetically altered mice showed that an intact main olfactory system is required for pup retrieval in lactating mice mothers (Weiss et al., 2011; Fraser and Shah, 2014), and that the VNO is necessary for the display of maternal aggression (Leypold et al., 2002; Hasen and Gammie, 2009; Chamero et al., 2011). Consequently, impairment of vomeronasal signaling in Trpc2^–/–^ lactating females has no impact on pup retrieval behavior, even though these females are deficient in maternal aggression (Leypold et al., 2002; Hasen and Gammie, 2009).

Yet, the VNO neuronal subpopulation implicated in maternal aggression is not characterized with certainty. While maternal aggression is nearly absent in basal VNO signaling-deficient cGαo^–/–^ females (Chamero et al., 2011), other studies have suggested some mediation from apical Gαi2/V1R-expressing VSNs (Del Punta et al., 2002; Norlin et al., 2003).

**FIGURE 2:**
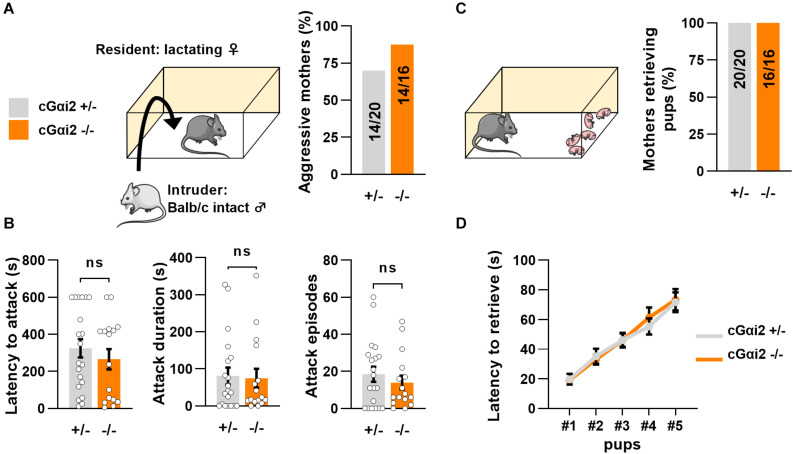
Loss of Gαi2 does not affect maternal behavior in lactating females. **(A)** Maternal aggression in lactating cGαi2^+/–^ (*N* = 20) vs. cGαi2^–/–^ (*N* = 16) females during three consecutive exposures to an intact male intruder in the resident-intruder test. 70% of cGαi2^+/–^ and 87% of cGαi2^–/–^ females present at least one aggressive episode (Fisher’s exact test: *P* = 0.3). **(B)** Measure of the aggression parameters latency to attack (*left*), cumulative attack duration (*middle*) and attack episodes (*right*) across genotypes (Mann-Whitney: *P* = 0.4–0.9). **(C)** 100% of cGαi2^+/–^ (*N* = 20) and cGαi2^–/–^ (*N* = 16) mothers retrieve five of their pups previously dispersed in the cage. **(D)** The latency to retrieve pups does not differ between groups (Dunn’s multiple comparison tests cGαi2^+/–^ vs. cGαi2^–/–^ for each pup’s latency *P* > 0.9).

Thus, we aimed to investigate the requirement of Gαi2^+^ vomeronasal neurons for parental behaviors in mated females. We first quantified territorial aggression of cGαi2^–/–^ vs. cGαi2^+/–^ lactating females in the resident-intruder paradigm (Figure 2). We observed a high number of animals attacking an adult intact male intruder in both cGαi2^–/–^ (14/16 mice; 87%) and cGαi2^+/–^ mothers (14/20 mice; 70%) in three consecutive 10 min tests (*P* = 0.3; Figure 2A). Attack latency, total attack duration, and cumulative attack numbers were high in both cGαi2^–/–^ and cGαi2^+/–^ females (*P* = 0.4–0.9; Figure 2B), indicating that Gαi2 is dispensable for the display of maternal aggression. Consistent with previous findings indicating a minor role of the VNO in the display of pup retrieval behavior in mothers (Wysocki and Lepri, 1991), cGαi2^–/–^ females exhibited normal pup retrieval when compared to heterozygous controls (Figures 2C,D). All females retrieved to the nest all of their pups randomly distributed in the home cage (Figure 2C), and the mean time to retrieve the 5 pups was less than 80 s in both groups (Figure 2D). Taken together, these results show that maternal aggression and pup retrieval by lactating females do not depend on Gαi2+ VSNs.

### Incomplete Sexual Receptivity Acquisition in Gαi2-Mutant Females

An intact VNO is required for the display of major female sexual behaviors, such as gender discrimination and sexual receptivity (Leypold et al., 2002; Stowers et al., 2002; Keller et al., 2006; Kimchi et al., 2007; Martel and Baum, 2009; Oboti et al., 2014; Hellier et al., 2018).

**FIGURE 3:**
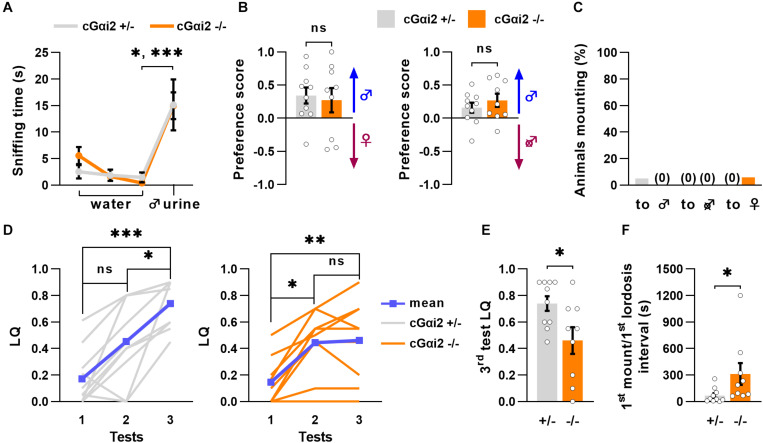
Incomplete sexual receptivity in Gαi2 mutant females. **(A)** cGαi2^+/–^ (*N* = 10) and cGαi2^–/–^ (*N* = 9) are able to detect intact male urine in the habituation—dishabituation paradigm (Kruskal-Wallis: *P* < 0.001, Dunn’s multiple comparison tests: water 3 vs. urine 1 cGαi2^+/–^ *P* < 0.05, cGαi2^–/–^ *P* < 0.001). **(B)** Olfactory preference of social cues in cGαi2^+/–^ (*N* = 10) and cGαi2^–/–^ (*N* = 9) female mice in a two-choice test. Females had the choice between male urine vs. estrus female (*left*) or castrated male (*right*) urine. cGαi2^+/–^ and cGαi2^–/–^ animals show a preference for male urine in both tests and no differences between genotypes are found (*t*-test: *P* = 0.4–0.7). **(C)** Ablation of Gαi2 in VSNs does not induce, in females, mounting behavior toward male, castrated male or female. The proportion of animals mounting is similar in cGαi2^+/–^ (*N* = 20) and cGαi2^–/–^ (*N* = 17) females (Fisher’s exact test: *P* = 0.5–0.9). **(D–F)** Sexual receptivity of cGαi2^+/–^ (*N* = 10) and cGαi2^–/–^ (*N* = 9) females during exposition to a sexually experienced stud male over three tests. **(D)** The lordosis quotient (LQ) of cGαi2^+/–^ (*left*) follows a normal increase over the tests (Kruskal-Wallis: *P* < 0.001, Friedman multiple comparisons: test 1 vs. 2 *P* = 0.5, test 1 vs. 3 *P* < 0.001, and test 2 vs. 3 *P* < 0.05), but in cGαi2^–/–^ (*right*) the lordosis quotient does not increase in the third test (Kruskal-Wallis: *P* < 0.001, Friedman multiple comparisons: test 1 vs. 2 *P* < 0.05, test 1 vs. 3 *P* < 0.01, and test 2 vs. 3 *P* = 0.9). **(E)** Comparison of the lordosis quotient of cGαi2^+/–^ and cGαi2^–/–^ females during the third test reveals a reduction of sexual receptivity in mutant females (Mann-Whitney: *P* < 0.05). **(F)** Latency to exhibit a lordosis, measured from the first mount received, is significantly longer in cGαi2^–/–^ females (Mann-Whitney: *P* < 0.05). *P < 0.05; **P < 0.01; ***P < 0.001.

To examine whether altered sexual behaviors of VNO-impaired mice might be related to a deficit in Gαi2 signaling, we measured olfactory preference to male odors in cGαi2^–/–^ females. We used adult, sexually naïve females subjected to ovariectomy and primed with estradiol and progesterone to minimize estrus cycle-related variability and increase their motivation for male pheromones. First, we evaluated their ability to recognize urine from an adult male using the habituation-dishabituation paradigm. cGαi2^–/–^ and cGαi2^+/–^ females were exposed to a sequence of three successive odor presentations of water and a final presentation of male urine. All females were able to recognize the urine, as the time spent sniffing the first urine presentation was higher than the previous water presentations (Figure 3A). Next, we analyzed the preference for either intact male urine or estrus female urine in a two-choice preference test. cGαi2^–/–^ and cGαi2^+/–^ females displayed the same level of preference for intact male urine (*P* = 0.8; Figure 3B). We then compared the preference for urine from either intact or castrated males (Figure 3B). cGαi2^–/–^ and cGαi2^+/–^ females showed a strong preference for intact male urine (*P* = 0.4; Figure 3B), indicating that preference for male odors is intact in cGαi2^–/–^ females.

Next, we assessed cGαi2^–/–^ females for the display of male-typical behaviors by scoring mounting behavior of intact female residents to either male, female, or castrated male intruders, independently introduced into the female resident’s home cage during a 10 min test period for 3 consecutive days. Very few animals displayed mounting behavior toward any intruder (female, male, or castrated male) for both cGαi2^–/–^ and cGαi2^+/–^ females. None of the cGαi2^–/–^ females exhibited mounting to male or castrated intruders, although some low levels of mounting were observed in 6% of females, to female intruders (Figure 3C). Thus, deletion of Gαi2 in VSNs does not enhance the display of male-specific behaviors in female mice.

We next asked whether normal olfactory preference and lack of mounting to males and females led to normal display of sexual receptivity in cGαi2^–/–^ females. To test this, we measured lordosis behavior, a female sexual stance held in response to male mounting that denotes sexual receptivity. Lordosis requires an intact VNO (Keller et al., 2006; Oboti et al., 2014; Hellier et al., 2018), and is significantly enhanced by experience (Thompson and Edwards, 1971; Bonthuis et al., 2011). Thus, we quantified lordosis response in the presence of a sexually experienced male in three tests separated by 4 days to mimic natural estrus occurrence. We observed low lordosis quotient (LQ; number of lordosis postures divided by the number of mounts received) values during the first test for all animals (LQ = 0.14–0.17; Figure 3D). The second test yielded an increase in LQ values of around 0.45, for both cGαi2^–/–^ and cGαi2^+/–^ females (Figure 3D). In the third test, however, cGαi2^–/–^ females remained at LQ levels similar to those of the second test (LQ = 0.46; Figure 3D), whereas cGαi2^+/–^ control females displayed a further LQ increase (from 0.45 to 0.74; Figure 3D). During the third test, each female received 20 mounts from the stud male, but cGαi2^–/–^ females responded displaying less lordosis than cGαi2^+/–^ controls (*P* < 0.05; Figure 3E). Furthermore, cGαi2^–/–^ females exhibited a significantly longer latency to show the first lordosis episode (67.4 ± 26.2 s for cGαi2^+/–^ females; 311.8 ± 123.1 s for cGαi2^–/–^; *P* < 0.05; Figure 3F). Taken together, these results indicate that Gαi2 vomeronasal neurons play a critical role in the acquisition of complete sexual receptivity in females, but not in olfactory preference and sexual partner choice.

### Reduced c-Fos Expression in the AOB and RP3V of Gαi2-Mutant Females

**FIGURE 4:**
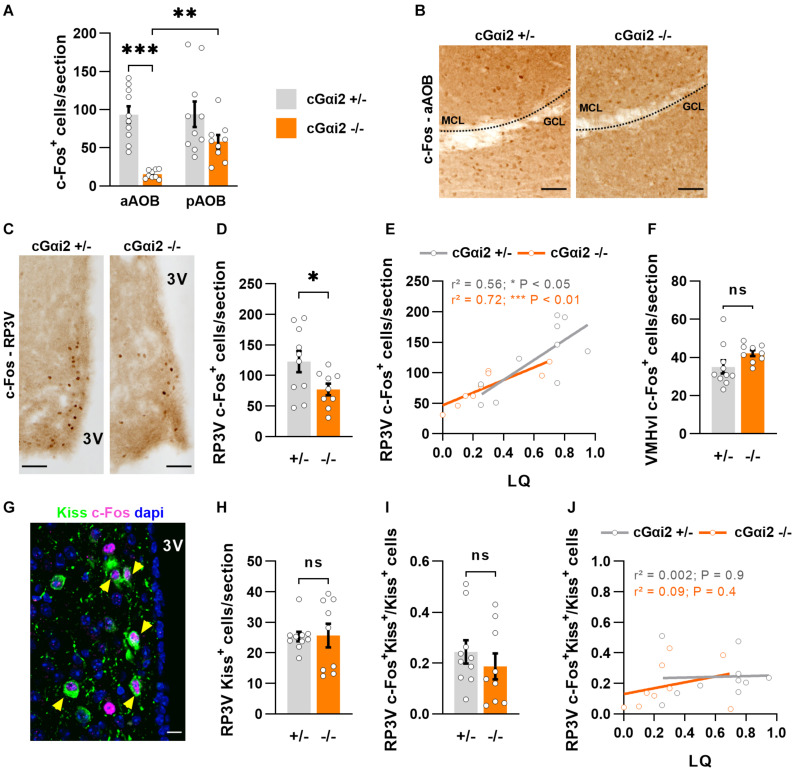
Reduced neural activity in the AOB and RP3V of Gαi2-mutant females. **(A,B)** Analysis of c-Fos immunodetection in the AOB of cGαi2^+/–^ (*N* = 10) and cGαi2^–/–^ (*N* = 9) females after sexual behavior. **(A)** Quantification of c-Fos+ cells per section reveals a decreased c-Fos activation in the aAOB of cGαi2^–/–^ [Two-way repeated measures ANOVA: (genotype) *F*_(__1, 17)_ = 18.28, *P* < 0.001; (region) *F*_(__1, 17)_ = 5.82, *P* < 0.05; (genotype × region) *F*_(__1, 17)_ = 5.53, *P* < 0.05; Bonferroni *post hoc* tests: cGαi2^–/–^ aAOB vs. cGαi2^–/–^ pAOB, *P* < 0.01; cGαi2^+/–^ aAOB vs. cGαi2^–/–^ aAOB *P* < 0.001; other comparisons *P* = 0.06–0.9). All three AOB layers were quantified. **(B)** Examples of c-Fos immunolabeling in the aAOB of cGαi2^+/–^ and cGαi2^–/–^females after sexual behavior. Scale bars, 50 μm. **(C–E)** c-Fos immunodetection in the RP3V of cGαi2^+/–^ (*N* = 10) and cGαi2^–/–^ (*N* = 9) females. **(C)** Examples of c-Fos immunodetection in the RP3V of cGαi2^+/–^ and cGαi2^–/–^females after sexual behavior. Scale bars, 50 μm. **(D)** Quantification of c-Fos+ cells per section in the RP3V of cGαi2^–/–^ (*t*-test: *P* < 0.05). **(E)** Positive correlation between the number of RP3V-c-Fos+ cells and lordosis quotient (LQ) in cGαi2^+/–^ and cGαi2^–/–^ females (Pearson’s correlation; cGαi2^+/–^: *r*^2^ = 0.56, *P* < 0.05; cGαi2^–/–^: *r*^2^ = 0.72, *P* < 0.01). **(F)** c-Fos immunodetection in the VMHvl of cGαi2^+/–^ (*N* = 10) and cGαi2^–/–^ (*N* = 9) females after sexual behavior. Quantification is not different between genotypes (*t*-test: *P* = 0.08). **(G–J)** Analysis of kisspeptin and c-Fos double immunostaining in the RP3V. **(G)** Example of Kiss and c-Fos immunostaining in the RP3V of a cGαi2^+/–^ female. The yellow arrows indicate double labeled c-Fos-Kiss neurons. Scale bar, 10 μm. Quantifications of Kiss+ neurons **(H)** and double-labeled c-Fos+Kiss+ neurons **(I)** are not different between genotypes (Mann-Whitney: *P* = 0.9; *t*-test: *P* = 0.4, respectively). **(J)** Correlative analysis between the number of c-Fos+Kiss+ cells in the RP3V and the LQ (Pearson’s correlation; cGαi2^+/–^: *r*^2^ = 0.002, *P* = 0.9; cGαi2^–/–^: *r*^2^ = 0.09, *P* = 0.4). AOB, accessory olfactory bulb (aAOB, anterior AOB; pAOB, posterior AOB); 3V, third ventricle; RP3V, rostral periventricular area of the third ventricle; VMHvl, ventrolateral part of the ventromedial hypothalamic nucleus; Kiss, kisspeptin. *P < 0.05; **P < 0.01; ***P < 0.001.

We further investigated neuronal activity after sexual behavior in downstream vomeronasal neural pathways in the brain. First, we quantified c-Fos expression in the AOB (Figures 4A,B). In control cGαi2^+/–^ females, the number of c-Fos+ cells in the AOB was elevated (∼100 cells/section) in both the anterior and posterior AOB (Figure 4A). By contrast, cGαi2^–/–^ females displayed significantly lower number of c-Fos+ in the anterior AOB (15 cells/section; *P* < 0.001; Figure 4A), while c-Fos+ cells in the posterior AOB was not significantly different from cGαi2^+/–^ controls (58 cells/section; *P* = 0.06; Figure 4A). This result suggests that Gαi2-independent VNO activity is sufficient for the display of basal levels of sexual receptivity.

We further asked whether this reduction of sensory information may have an impact on the neural activity of the rostral periventricular area of the third ventricle (RP3V), a hypothalamic region involved in the control of female sexual behavior and activated by male pheromones (Simerly, 2002; Bakker et al., 2010; Hellier et al., 2018; Inoue et al., 2019; Figures 4C–E). We thus counted c-Fos+ nuclei in the RP3V of the same females after sexual behavior. Consistent with a reduced sensory input, the density of c-Fos+ nuclei was lower in cGαi2^–/–^ females vs. cGαi2^+/–^ controls (*P* < 0.05; Figures 4C,D). To determine whether the extent of c-Fos+ cells in the RP3V was linked to sexual receptivity, we performed a correlation analysis between c-Fos cell density and LQ (Figure 4E). The number of c-Fos+ cells was positively correlated with the LQ in both genotypes (Figure 4E), suggesting that the level of sexual receptivity displayed may depend on RP3V neural activity. c-Fos activation in the ventrolateral part of the ventromedial hypothalamic nucleus (VMHvl), a region innervated by RP3V neurons and also involved in the control of female sexual behavior (Pfaff and Sakuma, 1979), revealed no significant differences (Figure 4F), suggesting that the observed alterations in cGαi2^–/–^ female receptivity are likely coded upstream of VMHvl neurons.

To further characterize in more detail the activated RP3V neurons in cGαi2^–/–^ females, we asked whether these cells belong to a neuronal subpopulation positive for the neuropeptide kisspeptin (Kiss), which is essential to trigger the lordosis response (Hellier et al., 2018; Figures 4G–J). Using double label immunostaining we found neurons that were positive for both c-Fos^+^ and Kiss in all animals (Figure 4G). We observed no difference in the density of Kiss^+^ neurons and the number of double-labeled c-Fos^+^/Kiss^+^ neurons in both cGαi2^–/–^ and cGαi2^+/–^ females (*P* = 0.4–0.9; Figures 4H,I). To establish whether the level of sexual receptivity is linked to the number of activated kisspeptin neurons in the RP3V, we performed a correlation analysis between c-Fos+/Kiss+ cell density and the LQ (Figure 4J). We did not observe any obvious correlation between the number of activated kisspeptin neurons and the LQ for any of the two genotypes (*r*^2^ = 0.002 and 0.09; Figure 4J), suggesting that an increase in sexual receptivity does not require the recruitment of additional kisspeptin neurons. Collectively, these results indicate that Gαi2 vomeronasal signaling is dispensable for the display of basal levels of lordosis, but participates in the acquisition of complete sexual receptivity, possibly through RP3V kisspeptin-negative neurons.

### Gαi2 Deletion Has No Impact on Reproductive Physiology

Reduced receptivity to males could be a consequence of not only defective VNO chemodetection but also of altered reproductive physiology. Indeed, females with a total or partial ablation of VNO function (Trpc2^–/–^ and cGαo^–/–^ females) exhibit profound alterations in puberty onset and estrus cyclicity, even in the absence of external stimuli (Flanagan et al., 2011; Oboti et al., 2014).

Therefore, as a further control and to verify that the described loss of functions by the conditional Gαi2 ablation are indeed caused by a loss of VNO signaling and not by defective reproductive physiology, we investigated the impact of Gαi2 deletion on the timing of estrus and ovulation. First, we characterized puberty onset of juvenile female mice. Control cGαi2^+/–^ and cGαi2^–/–^ females were examined for the onset of vaginal opening and first estrus. Animals from both genotypes displayed vaginal opening around postnatal day 35 (*P* = 0.6; Table 1) and first estrus a day later (*P* = 0.5; Table 1), indicating normal puberty onset in Gαi2 mutants. Next, we analyzed estrus cycles of adult, group-housed female mice during a 2 week interval. Both control cGαi2^+/–^ and cGαi2^–/–^ females displayed consistent estrus cycles with no difference in the mean cycle duration (*P* = 0.1; Table 1) and similar number of proestrus and estrus days (*P* = 0.5; Table 1). Consistent with normal ovarian function, levels of circulating progesterone during the follicular or luteal phases (*P* = 0.2 and 0.9; Table 1), and average ovarian weight were not different between cGαi2^–/–^ and cGαi2^+/–^ females (*P* = 0.2–0.9; Table 1). We measured uterus weight as a proxy for circulating estradiol, and found a weight decrease during the luteal vs. follicular phases (*P* < 0.001 and 0.05; Table 1), but no differences between genotypes (*P* = 0.2–0.9; Table 1). Finally, we compared three fertility parameters—percentage of pregnant females after 7 days, delivery latency and litter size—and found no differences between cGαi2^–/–^ and cGαi2^+/–^ (*P* = 0.2–0.3; Table 1). Together, these findings indicate that reproductive physiology and ovarian function are normal in cGαi2^–/–^ females.

**TABLE 1:** cGαi2^–/–^ females show normal reproductive physiology.

		**cGα i2^+/^**^–^	**cGα i2**^–^**^/^**^–^	**Statistic**	***P*-value**	
**Puberty**						
	Age at vaginal opening (days)	34.6 ± 0.8 (17)	35.1 ± 0.8 (15)	Unpaired *t-*test	0.6	*ns*
	Age at first estrus (days)	36.2 ± 0.7 (17)	36.8 ± 0.6 (15)	Unpaired *t-*test	0.5	*ns*
**Estrus cycle**						
	Cycle length (days)	4.9 ± 0.1 (47)	5.5 ± 0.2 (47)	Mann-Whitney	0.1	*ns*
	Proestrus + estrus days	6.6 ± 0.2 (47)	6.4 ± 0.2 (45)	Mann-Whitney	0.5	*ns*
**Serum steroids levels**						
Progesterone (ng.mL^–1^)	Follicular phase	3.2 ± 0.7 (10)	5.6 ± 1.3 (7)	Mann-Whitney	0.2	*ns*
	Luteal phase	7.9 ± 1.2 (36)	7.9 ± 1.5 (26)	Mann-Whitney	0.9	*ns*
**Reproductive organs weight**						
Ovaries (%bw)		0.033 ± 0.001 (27)	0.034 ± 0.002 (31)	Mann-Whitney	0.4	*ns*
Uterus (%bw)	Follicular phase	0.29 ± 0.02 (25)	0.32 ± 0.03 (21)	2*Kruskal-Wallis	0.9	*ns*
	Luteal phase	0.23 ± 0.02 (19) †	0.2 ± 0.01 (28) ‡		0.2	*ns*
**Fertility**						
	Pregnant females (%)	67/76 (88%)	54/67 (81%)	Fisher’s exact test	0.2	*ns*
	Delivery latency (days)	22.4 ± 0.3 (76)	23.3 ± 0.4 (67)	Mann-Whitney	0.2	*ns*
	Litter size	7.8 ± 0.4 (76)	7.1 ± 0.5 (67)	Mann-Whitney	0.3	*ns*

*† and ‡, respectively, P < 0.05 and P < 0.001 vs. the uterus weight during the follicular phase. bw, body weight. Assessment of puberty onset, estrus cyclicity, sex steroids levels, reproductive organs weight, and fertility parameters of control and cGαi2^–/–^ females. Values are means ± SEM of the indicated number (n) of females.*

## Discussion

The central hypothesis of this study was that detection of environmental stimuli by the VNO modulates experience-dependent plasticity, possibly influencing the display of social behaviors in female mice. To test this hypothesis, we chose an animal model with a conditional mutation for Gαi2 gene, which harbors a partial loss of VNO function (Trouillet et al., 2019), and two experimental paradigms (pup retrieval and sexual receptivity) that are strongly dependent on social experience (repeated contact with pups or the male). We previously showed that Gαi2 is necessary for the detection of small organic pheromones by apical VSNs, and genetic ablation of these neurons severely impairs pup-directed aggression in virgin males (Trouillet et al., 2019). This is consistent with published studies that show that pup cues preferentially activate neurons located in the apical VNO and the anterior AOB in virgin males (Tachikawa et al., 2013; Nakahara et al., 2020). Our results show higher c-Fos activation in the anterior AOB after pup-exposure (Figures 1C,D), indicating that pups are also preferentially detected by Gαi2+ VSNs in virgin females.

In contrast to males, we found that deletion of Gαi2 in virgin females does not increase parental care (i.e., grooming) toward pups, but reduces pup retrieval (Figures 1A,B). Interestingly, mutant females that retrieve pups still display other types of parenting behaviors such as grooming and nesting, suggesting that different pup care behaviors are controlled independently. Importantly, Gαi2 deletion does not affect pup retrieval and maternal aggression in mothers (Figure 2), indicating that other neural substrates different from Gαi2^+^ VSNs control these behaviors, likely by auditory, olfactory and Gαo-dependent signals (Chamero et al., 2011; Weiss et al., 2011; Fraser and Shah, 2014; Marlin et al., 2015).

The mechanism by which social experience drives neural plasticity to modify the behaviors display is unclear. Not only centrally controlled neural changes, but also sensory organ sensitivity to olfactory stimuli have been suggested to have an influence. In particular, previous studies report a decline in VNO activity induced by pup cues in virgin males after cohabitation with a pregnant female (Tachikawa et al., 2013; Nakahara et al., 2020), suggesting the existence of a silencing mechanism at the level of VSNs. We can thus speculate that a similar process may occur in females after parturition, either induced by hormonal changes intrinsic to gestation, or by repeated exposure to pups. Indeed, pup retrieval is improved in virgin females after co-housing with a dam and litter, even after ovariectomy (Ehret et al., 1987; Koch and Ehret, 1989; Stolzenberg and Rissman, 2011; Elyada and Mizrahi, 2015), suggesting that pup signals modulate the behavioral response. In this scenario, hormones such as oxytocin may influence experience-dependent plasticity directly at the sensory processing level (Fleming et al., 1979; Marlin et al., 2015; Nakahara et al., 2020).

Studies on Trpc2^–/–^ mice suggest that sex discrimination and opposite-sex preference depend on a functional VNO because of indiscriminate mounting expressed by males and females (Leypold et al., 2002; Stowers et al., 2002; Kimchi et al., 2007). Other studies point to a significant role of testosterone levels (rather than VNO function) to explain the unusual mounting of Trpc2^–/–^ females (Martel and Baum, 2009). The absence of mounting behavior in our Gαi2 mutant females (Figure 3C) as well as in conditional Gαo mutants (Oboti et al., 2014), suggest a minor role of the VNO in the control of sexual preference in females. Nonetheless, either the apical or basal VSN layers alone may be sufficient to enable sex preference by using redundant neural pathways (Chamero et al., 2012). Further research is required to elucidate the control of sexual preference.

Although many aspects of sexual receptivity in females may be innate, acquiring elevated levels of lordosis performance depends on the combination of adequate hormonal environment with sensory experience (Thompson and Edwards, 1971). It has been shown that lesions of the VNO and AOB reduce the lordosis quotient of sexually naïve females (Keller et al., 2006; Martel and Baum, 2009) and that acute inhibition of AOB activity reduces the lordosis quotient of sexually experienced females (McCarthy et al., 2017). Our results show that Gαi2-dependent VNO activity is dispensable for the display of basal levels of lordosis (Figure 3), which likely depends on Gαo signaling (Oboti et al., 2014). After mating experience, however, we demonstrate that Gαi2+ VSNs play a critical role in the acquisition of complete sexual receptivity in females, transforming weaker responses into more robust and frequent lordosis episodes (Figure 3). Thus, synergy between innate and experience-dependent processes may be critical for fast, efficient, and flexible display of complex behaviors.

We have screened for the expression of c-Fos, an early gene linked to neural activity, in the brain and found that the number of c-Fos+ cells was reduced in the anterior AOB and RP3V of cGαi2^–/–^ females after lordosis (Figure 4). This reduction in c-Fos+ neurons in both regions is consistent with reduced sensory input from the VNO. However, we observe a positive correlation of the number of c-Fos+ cells in the RP3V with lordosis, also in control animals (Figure 4E). Further experiments are needed to establish causality, but these results suggest that neural activity in the RP3V may regulate the level of sexual receptivity integrating sensory information from Gαi2+ neurons. In particular, RP3V-kisspeptin neurons seem to be essential to trigger lordosis (Hellier et al., 2018), although activity of these cells is not lower in cGαi2^–/–^ females (Figure 4I). This is in line with the view that the display of basal levels of lordosis is largely Gαi2-independent and suggests that the recruitment of additional RP3V-kisspeptin neurons is not required to increase lordosis performance. In accordance with this, non-kisspeptin neurons in the RP3V, such as tyrosine-hydroxylase (TH) positive neurons, are also sensitive to male odors (Bakker et al., 2010; Taziaux and Bakker, 2015). Remarkably, targeted ablation of TH neurons in the RP3V does not seem to reduce the lordosis quotient in sexually naïve females (Scott et al., 2015). Further research is needed to establish whether TH neurons in the RP3V participate in the acquisition of sexual experience.

**FIGURE 5:**
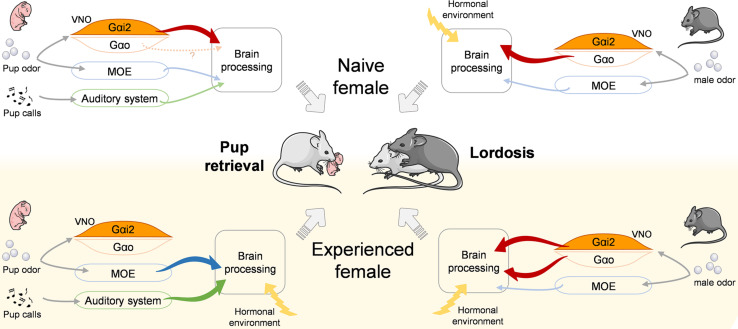
Model for the proposed control of pup retrieval and lordosis behaviors depending on social experience. Olfactory and auditory cues relevant for pup retrieval (***left***) and lordosis (***right***) behaviors are differentially processed by the sensory systems and the brain in naïve (***top***) and experienced (***down***) females. Significantly, Gαi2+ vomeronasal neurons are activated by pup odor in naïve females to trigger retrieval but are dispensable in experienced females. Inversely, they are dispensable in naïve females to trigger the lordosis response but become necessary in experienced females to reach high lordosis levels. VNO, vomeronasal organ; MOE, main olfactory epithelium.

Our findings complement recent studies of neural circuits involved in social behavior by revealing that experience-dependent improvement of ethologically important behaviors can be shaped by sensory components (Figure 5). This may exemplify a mechanism of neuroplasticity in which olfaction in combination with social experience improve social behavior synergistically.

## Data Availability Statement

The raw data supporting the conclusions of this article will be made available by the authors, without undue reservation.

## Ethics Statement

The animal study was reviewed and approved by the CEEA Val de Loire.

## Author Contributions

PC, MK, LB, FZ, and TL-Z designed the research. A-CT, CM, and KP performed the research. A-CT and CM analyzed the data. PC and A-CT wrote the manuscript with edits of all authors.

## Conflict of Interest

The authors declare that the research was conducted in the absence of any commercial or financial relationships that could be construed as a potential conflict of interest.
